# Sociodemographic factors associated with depressive symptoms and self-care agency capacity in hypertensive older adults from Loja, Ecuador: a cross-sectional study

**DOI:** 10.3389/fpubh.2026.1888008

**Published:** 2026-09-10

**Authors:** Eddison Josué Ramírez-Merchán, María José Mendoza Sarango, Valeria Katherine Quizhpi Guachizaca, Jefersson Santos-Yate, Alexander Casallas-Vega, Jeisson Andrés Hincapié-Carvajal

**Affiliations:** 1 Departamento de Ciencias de la Salud, Carrera de Enfermería, Grupo de Investigación Enfermería, Contextos y Realidades, Universidad Técnica Particular de Loja, Loja, Ecuador; 2 Centro de Investigación e Innovación, Subred Integrada de Servicios de Salud Sur E.S.E, Bogotá, Colombia; 3 Vicerrectoría de Investigaciones, Universidad FUCS, Bogotá, Colombia; 4 Programa de Enfermería, Grupo de Investigación en Salud Pública, Escuela de Medicina y Ciencias de la Salud, Universidad del Rosario, Bogotá, Colombia

**Keywords:** aging, depression, hypertension, self care, social determinants of health

## Abstract

**Background:**

Hypertension is one of the leading chronic diseases affecting older adults and is frequently associated with depressive symptoms and difficulties in self-care. Sociodemographic conditions may influence both mental health and self-care behaviors; however, evidence in older hypertensive populations from Ecuador remains limited. Therefore, this study aimed to analyze the association between sociodemographic factors, depressive symptoms, and self-care agency capacity among older adults with hypertension living in Loja, Ecuador.

**Methods:**

A cross-sectional study was conducted among 222 older adults diagnosed with hypertension residing in urban, rural, and urban-marginal areas of Loja, Ecuador. Depressive symptoms were assessed using the Beck Depression Inventory-II (BDI-II), while self-care agency capacity was evaluated using the Self-Care Agency Capacity Scale for Patients with Hypertension. Descriptive analyses, Spearman correlation, bivariate analyses, and multiple linear regression models were performed, adjusted for sociodemographic variables.

**Results:**

Most participants showed high self-care agency capacity (69.4%), whereas 84.2% presented minimal depressive symptoms. After adjustment, place of residence remained independently associated with self-care agency capacity, with participants living in urban-marginal areas showing higher self-care agency capacity scores than those residing in rural areas. Depressive symptoms were significantly associated with sex, age and place of residence. No statistically significant correlation was observed between depressive symptoms and self-care agency capacity (rho = 0.111; *p* = 0.100).

**Conclusion:**

Sociodemographic factors were associated with both depressive symptoms and self-care agency capacity among older adults with hypertension. These findings highlight the importance of considering social determinants and mental health conditions in the comprehensive management of chronic diseases in older populations.

## 1 Introduction

The increase in life expectancy and population aging has generated new challenges for healthcare systems, particularly in low- and middle-income countries. Since 2020, the global population aged over 60 years has, for the first time, exceeded the number of children under 5 years of age, and it is estimated that by 2030, one in six people worldwide will belong to this age group (1). In this context, arterial hypertension (HTN) represents a major public health problem and one of the leading causes of cardiovascular morbidity and mortality worldwide. Its increasing prevalence has been associated with population aging, urbanization, and various social determinants of health that act synergistically (2, 3). Furthermore, among older adults, HTN is associated with an increased risk of ischemic heart disease, heart failure, cerebrovascular disease, chronic kidney disease, and cognitive impairment (4, 5), thereby increasing the economic and healthcare burden in this population, with estimated annual healthcare costs of approximately USD 2,453 per person (6).

In Ecuador, HTN represents one of the leading chronic conditions affecting older adults, with a pooled prevalence of 35.8% in the adult population and approximately 48.7% among individuals older than 60 years. Furthermore, a study conducted in older adults from Loja reported that more than half of the participants (53.7%) had HTN, highlighting the substantial burden of this condition in the province (7, 8).

Beyond its physical consequences, HTN frequently coexists with mental health disorders, particularly depression, which represents one of the main mental health conditions affecting older adults. Globally, it is estimated that approximately 14% of individuals over 60 years of age experience some type of mental disorder, with depression and anxiety being the most common conditions (9). In Latin America, the prevalence of depressive disorders reaches 5.3% within a 12-month period and 12.5% across the lifetime, with variations associated with social inequalities and levels of human development (10).

In this context, HTN has been identified as a factor associated with the development of depressive symptoms in older age (11), and a high frequency of comorbidity between both conditions has also been observed in geriatric populations (12). According to the “vascular depression” hypothesis, HTN and other cardiovascular risk factors may contribute to the onset or persistence of depressive symptoms through cerebrovascular alterations and dysfunction of the frontostriatal circuits involved in emotional regulation (13). Despite the documented burden of HTN and its association with depressive symptoms, regional evidence examining the factors associated with HTN and its relationship with mental health and self-care remains limited.

Given the chronic nature of HTN, self-care constitutes a fundamental component in disease control and the prevention of cardiovascular complications. Among HTN older adults, self-care behaviors have been associated with better therapeutic adherence, blood pressure control, and quality of life (14). According to Orem’s Self-Care Deficit Nursing Theory, individuals are active agents responsible for maintaining their own health through self-care activities (15). Self-care agency capacity (SCAC) refers to the acquired ability to engage in self-care activities, which is influenced by Basic Conditioning Factors, including age, sex, health status, sociocultural context, developmental stage, life experiences, and environmental conditions (16, 17). In older adults with HTN, these factors may affect adherence to treatment, lifestyle modification, symptom monitoring, and long-term disease management, making SCAC a key construct for understanding disease management and health outcomes in older adults with HTN. Previous studies have shown that SCAC and self-care behaviors among individuals with HTN may be influenced by sociodemographic characteristics, as well as psychosocial factors such as family support, illness perception, and treatment-related characteristics (16, 18, 19).

Despite the relevance of HTN in older adult populations, SCAC and depressive symptoms have generally been examined separately, and evidence regarding their joint assessment remains limited (20, 21). Likewise, regional evidence is scarce, and, to the best of our knowledge, no studies have jointly examined SCAC and depressive symptoms among older adults with HTN in Ecuador. This knowledge gap limits the development of comprehensive strategies aimed at strengthening both self-care and mental health in this population.

Therefore, this study aimed to analyze the association between sociodemographic factors, depressive symptoms, and SCAC among older adults with HTN living in Loja Province, Ecuador. This province is characterized by marked population heterogeneity, encompassing urban, rural, and urban-marginal areas, which provides a relevant setting for examining the influence of social determinants on mental health and self-care among hypertensive older adults. By addressing these relationships, this study contributes to the United Nations Sustainable Development Goal (SDG) 3 (Good Health and Well-being), particularly Target 3.4, which seeks to reduce the burden of non-communicable diseases while promoting mental health and well-being. We hypothesized that sociodemographic factors would be independently associated with both depressive symptoms and self-care agency capacity among older adults with arterial hypertension.

## 2 Materials and methods

### 2.1 Study design and setting

An observational, analytical, cross-sectional study was conducted to analyze the association between sociodemographic factors, depressive symptoms, and SCAC among older adults with HTN. The study was carried out in Loja Province, located in southern Ecuador, between September 2024 and February 2025.

Loja Province presents heterogeneous geographic and social characteristics, including urban, rural, and urban-marginal areas, which allowed the inclusion of participants from different territorial contexts. The study was reported following the recommendations of the Strengthening the Reporting of Observational Studies in Epidemiology (STROBE) guidelines for observational studies (22).

### 2.2 Population, sample, and sampling

The study population consisted of 13,380 older adults estimated from the 57,799 older adults reported in Loja Province (23), considering a theoretical combined prevalence of HTN and depressive symptoms of 23.15% in this age group (24).

Sample size was calculated using the formula for finite populations, considering a 95% confidence level and a 5% margin of error. The expected proportion (*p*) was established at 23.15% and its complement (*q*) at 76.85%, resulting in a representative sample of 268 participants. A non-probability convenience sampling method was employed because probability sampling was not feasible due to the lack of updated records for eligible participants in some health centers and the frequent mobility of the older adult population, which limited the construction of a reliable sampling frame.

The inclusion criteria were: (a) individuals aged 65 years or older; (b) a previous clinical diagnosis of HTN documented in the primary healthcare records of the Ministry of Public Health of Ecuador and receiving follow-up in health centers in the city of Loja; and c) voluntary acceptance to participate through signed informed consent.

Individuals who did not meet the eligibility criteria were not enrolled. In addition, participants were excluded if they: (a) discontinued questionnaire completion; (b) declined to participate in the study; or (c) presented cognitive or comprehension difficulties that prevented the adequate completion of the instruments.

Although the minimum required sample size was 268 participants, approximately 350 potentially eligible individuals were approached through home visits, and the final analytical sample consisted of 222 participants after applying the eligibility criteria and excluding participants with incomplete data. To assess whether the final sample remained adequate for the planned multiple linear regression analyses, a sensitivity analysis was performed using G*Power (version 3.1.9.7) (25). Considering 11 predictors, an alpha level of 0.05, and a statistical power of 95%, the analysis indicated that the final sample could detect a minimum effect size of *f*^2^ = 0.119, corresponding to an effect between small and medium according to Cohen’s criteria. Therefore, the final analytical sample was considered adequate for the planned regression analyses.

### 2.3 Recruitment procedure

Participants were identified from healthcare records of older adults diagnosed with HTN receiving care in primary healthcare units of the Ministry of Public Health in Loja Province. Recruitment was conducted through home visits performed by researchers and primary healthcare personnel.

**Figure 1:**
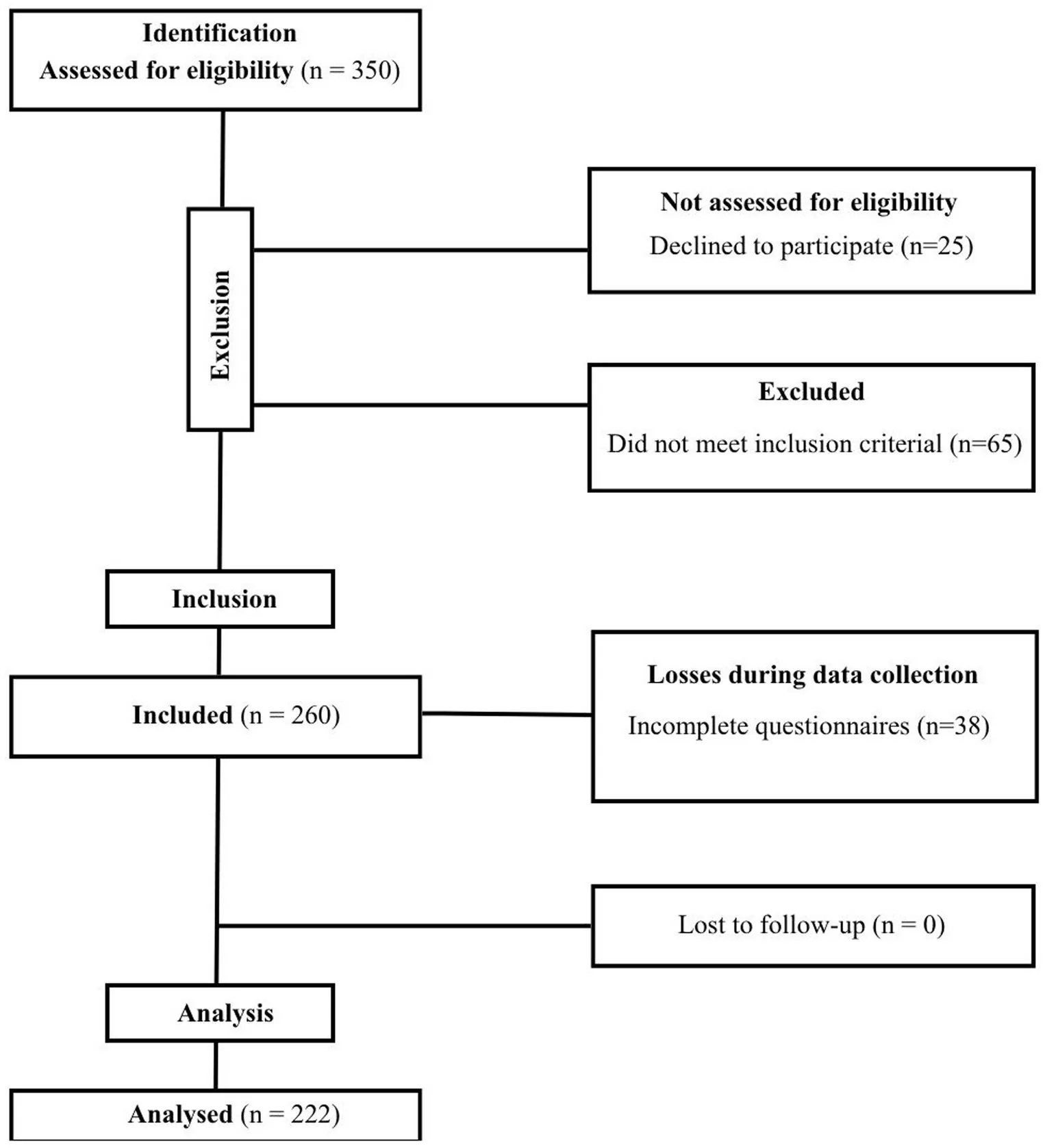
Flowchart of participant selection and recruitment.

In order to achieve the estimated sample size and considering potential losses during community fieldwork, approximately 350 home visits were conducted. After applying the eligibility criteria and excluding participants who declined participation or did not complete the instruments, the final sample consisted of 222 older adults. The main cause of participant loss was related to low interest in participating during the data collection process (see Figure 1).

### 2.4 Instruments

Data collection was conducted using a structured questionnaire composed of three sections: (a) sociodemographic variables; (b) self-care agency capacity; and (c) depressive symptoms.

The first section included an eight-item questionnaire designed by the researchers to collect information regarding age, sex, educational level, marital status, place of residence, ethnicity, and family structure. Educational level was categorized as Basic education (complete or incomplete primary education), intermediate education (complete or incomplete secondary education), and higher education (complete higher education).

Self-care agency capacity was assessed using the Self-Care Agency Capacity Scale for Patients with Arterial Hypertension, developed and validated by Achury et al. (26). The instrument has been used in several studies conducted in hospital and community settings among Colombian populations (27, 28). The questionnaire consists of 17 items distributed across three dimensions: fundamental capabilities and willingness for self-care (3 items), power component (4 items), and operational self-care capacity (10 items). Responses were recorded by selecting a single response on a five-point Likert scale ranging from 1 (“never”) to 5 (“always”), with total scores ranging from 17 to 85 points. Due to the negative directionality of some items, scores for items 8, 16, and 17 were reverse-coded before calculating the global score. Higher scores reflected greater self-care capacity. According to the classification proposed by the authors, the global score was categorized as low capacity (17–28 points), moderate capacity (29–56 points), and high capacity (57–85 points).

The original instrument reported adequate internal consistency, with a Cronbach’s alpha coefficient of 0.75 (26). In the present study, the questionnaire demonstrated high internal consistency, with an overall Cronbach’s alpha of 0.86.

Depressive symptoms were assessed using the Beck Depression Inventory-II (BDI-II), developed by Beck et al. (29). This instrument has been widely used to assess depressive symptomatology in adult and older adult populations due to its adequate psychometric validity and ease of administration. The BDI-II consists of 21 items that assess cognitive, affective, motivational, and somatic symptoms of depression. Each item is scored on a four-point scale ranging from 0 to 3, and participants were instructed to select the single statement that best described how they had felt during the previous 2 weeks. Higher total scores indicated greater severity of depressive symptoms.

Studies conducted in different settings have reported Cronbach’s alpha coefficients ranging from 0.80 to 0.93, demonstrating adequate internal consistency of the instrument (30, 31). In the present study, the BDI-II obtained a Cronbach’s alpha of 0.88.

The severity of global depressive symptoms was classified according to the instrument’s established cut-off points: minimal depression (0–13 points), mild (14–19 points), moderate (20–28 points), and severe (≥29 points) (see Supplementary file 1).

### 2.5 Administration procedure

The questionnaire was administered digitally during home visits conducted by the research team in collaboration with primary healthcare personnel between September 2024 and February 2025. Data collection was organized to avoid interfering with routine community healthcare activities. The average response time was approximately 15 min.

### 2.6 Statistical analysis

Data were processed using Jamovi (32, 33), an open-access statistical package based on the R programming language. Descriptive analyses were performed using means and standard deviations for quantitative variables, as well as absolute frequencies and percentages for categorical variables. Data distribution was evaluated using the Kolmogorov–Smirnov normality test. Depressive symptom scores did not show a normal distribution (*p*-values ranging from 0.006 to <0.001), whereas SCAC scores demonstrated normal distribution (*p*-values ranging from 0.176 to 0.643).

According to the distribution of the variables, both parametric and non-parametric tests were applied. For variables with normal distribution, Student’s *t*-test and ANOVA were used, whereas Mann–Whitney *U* and Kruskal–Wallis tests were applied for non-parametric variables.

The association between depressive symptoms and SCAC was evaluated using Spearman’s correlation due to the non-normal distribution of at least one of the study variables. Additionally, multiple linear regression analyses were performed to identify the association between sociodemographic factors, depressive symptoms, and SCAC. Incomplete questionnaires were excluded from the analysis. A statistical significance level of *p* < 0.05 was considered.

### 2.7 Ethical considerations

The study was approved by the Ethics Committee of the Universidad Técnica Particular de Loja (UTPL), Ecuador, under code 2024-04-INT-EO-RM-004. All participants signed informed consent prior to data collection. Information was anonymized before analysis and stored in a password-protected database accessible only to the principal investigator.

## 3 Results

### 3.1 Sociodemographic characteristics of the sample

A total of 222 hypertensive older adults were included in the analysis. The mean age of the participants was 73.9 ± 6.56 years, of whom 55.9% (*n* = 124) belonged to the 65–74 years age group, followed by those aged 75–84 years with 36.9% (*n* = 82). Female participants represented 59.5% (*n* = 132) of the sample, and the predominant marital status was married 63.5% (*n* = 141). Regarding educational level, 82.4% (*n* = 183) had a basic level of education. Distribution according to place of residence included participants from urban areas 40.5% (*n* = 90), rural areas 39.2% (*n* = 87), and urban-marginal areas 20.3% (*n* = 45). The predominant family structure was the nuclear family (63.1%) (see Table 1).

**Table 1:** Sociodemographic characteristics and comparison of depression and self-care scores among hypertensive older adults (*n* = 222).

Variable	*N* (%)	Self-care agency capacity	Statistic	*p*	Depressive symptoms (BDI-II)	Statistic	*p*
		Mean (±SD)			Mean (±SD)		
Age (mean ± SD)							
65–74 years	124 (55.9)	58.70 ± 10.6	*F* = 1.89	0.163^a^	5.85 ± 5.67	*H* = 22.3	<0.001^c^
75–84 years	82 (36.9)	61.30 ± 11.4			6.80 ± 6.47		
≥85 years	16 (7.2)	56.8 ± 10.2			16.63 ± 10.03		
Marital status							
Married	141 (63.5)	59.2 ± 10.9	*t* = −1.07	0.284^b^	5.79 ± 6.26	*U* = 3,965	<0.001^d^
Living alone	81 (36.5)	60.1 ± 11.0			9.04 ± 7.49		
Sex							
Female	132 (59.5)	59.0 ± 10.9	*t* = −0.856	0.393^b^	8.30 ± 7.66	*U* = 4,429	0.001^d^
Male	90 (40.5)	60.3 ± 10.9			5.04 ± 5.03		
Educational level							
Basic education	183 (82.4)	58.8 ± 11.10	*F* = 4.99	0.013^a^	7.25 ± 7.32	*H* = 0.26	0.875^c^
Intermediate education	17 (7.7)	61.0 ± 9.03			5.73 ± 4.53		
Higher education	22 (9.9)	65.1 ± 10.17			5.65 ± 3.92		
Place of residence							
Rural	87 (39.2)	54.4 ± 14.49	*F* = 36.4	<0.001^a^	4.31 ± 5.79	*H* = 33.4	<0.001^c^
Urban	90 (40.5)	60.3 ± 9.25			7.97 ± 6.34		
Urban-marginal areas	45 (20.3)	67.7 ± 9.61			10.16 ± 8.09		
Religion							
Catholic	221 (99.5)	62.6 ± 12.5	*t* = −0.985	0.326^b^	6.99 ± 6.91	*U* = 86.5	0.712^d^
Christian	1 (0.5)	75.00 ± —			4.00 ± —		
Ethnicity							
White	3 (1.4)	52.3 ± 26.24	*F* = 4.97	0.164^a^	6.67 ± 7.02	*H* = 5.59	0.061^c^
Indigenous	2 (0.9)	56.5 ± 6.36			26.00 ± 2.83		
Mestizo	217 (97.7)	59.6 ± 10.91			6.81 ± 6.69		
Family structure							
Extended family	34 (15.3)	59.1 ± 11.4	*F* = 0.051	0.984^a^	7.79 ± 6.69	*H* = 14.0	0.003^c^
Single-parent family	24 (10.8)	59.3 ± 10.7			8.42 ± 6.63		
Nuclear family	140 (63.1)	59.4 ± 10.4			5.86 ± 6.43		
Single-person household	24 (10.8)	61.0 ± 14.0			10.92 ± 8.45		

a One-way ANOVA test.

b Student’s *t*-test.

c Kruskal–Wallis test.

d Mann–Whitney *U* test.

Regarding depressive symptoms, statistically significant differences were observed according to age, marital status, sex, place of residence, and family structure. Participants older than 85 years presented the highest mean depressive symptom scores compared with the other age groups (16.63 ± 10.03; *p* < 0.001). Likewise, participants living alone showed higher depressive symptom scores (9.04 ± 7.49; *p* < 0.001) compared with married participants.

Regarding sex, female participants reported higher depressive symptom scores compared with male participants (8.30 ± 7.66 vs. 5.04 ± 5.03; *p* = 0.001). Similarly, participants living in urban-marginal areas presented higher depressive symptom scores (10.16 ± 8.09; *p* < 0.001). Family structure also showed a significant association with depressive symptoms (*p* = 0.003), with participants from single-person households presenting the highest depressive symptom scores (10.92 ± 8.45).

Concerning SCAC, no statistically significant differences were found according to age, marital status, sex, religion, ethnicity, or family structure. However, place of residence showed a significant association with SCAC (*p* < 0.001). Participants residing in urban-marginal areas presented the highest SCAC scores (67.7 ± 9.61), followed by those living in urban areas (60.3 ± 9.25) and rural areas (54.4 ± 14.49). Educational level also showed a statistically significant association with SCAC (*p* = 0.013), with higher mean scores observed among participants with higher education (see Table 1).

### 3.2 Distribution of depressive symptoms and self-care agency capacity

A total of 84.2% (*n* = 187) of participants presented minimal depression levels, followed by mild depression in 10.8% (*n* = 24). Regarding SCAC, 69.4% (*n* = 154) showed high SCAC, 30.2% (*n* = 67) presented moderate capacity, and only 0.5% (*n* = 1) demonstrated low SCAC. Overall, the mean Beck Depression Inventory-II score was 6.98 ± 6.89 points, whereas the mean SCAC score was 59.5 ± 10.9 points (see Table 2).

**Table 2:** Distribution of depression levels and self-care agency capacity among hypertensive older adults.

Variables	General categories	Frequency
Self-care agency capacity	Low capacity	1 (0.5)
	Moderate capacity	67 (30.2)
	High capacity	154 (69.4)
Beck Depression Inventory-II	Minimal depression	187 (84.2)
	Mild depression	24 (10.8)
	Moderate depression	9 (4.1)
	Severe depression	2 (0.9)

Overall mean Beck Depression Inventory-II score = 6.98 ± 6.89; overall mean self-care agency capacity score = 59.5 ± 10.9.

### 3.3 Association between depressive symptoms and self-care agency capacity

Spearman’s correlation analysis did not show a statistically significant association between depressive symptoms and SCAC among the hypertensive older adults evaluated (*ρ* = 0.111; *p* = 0.100).

Multiple linear regression models were statistically significant for both SCAC and depressive symptoms. The multiple linear regression model for SCAC explained 25% of the variance (*R* = 0.500; adjusted *R*^2^ = 0.211; *F*(11, 210) = 6.36; *p* < 0.001). In this model, place of residence was the only variable independently associated with SCAC. Compared with participants living in urban areas, those residing in rural areas had significantly lower SCAC scores (*B* = −5.626, *β* = −0.514, *p* < 0.001), whereas those living in urban-marginal areas showed significantly higher SCAC scores (*B* = 8.207, *β* = 0.750, *p* < 0.001).

The multiple linear regression model for depressive symptoms explained 31.3% of the variance (*R* = 0.559; adjusted *R*^2^ = 0.277; *F*(11, 210) = 8.68; *p* < 0.001). Male sex was independently associated with lower depressive symptom scores compared with female sex (*B* = −2.255, *β* = −0.327, *p* = 0.011). Participants aged ≥85 years presented significantly higher depressive symptom scores than those aged 65–74 years (*B* = 8.593, *β* = 1.246, *p* < 0.001). In addition, participants living in rural areas had significantly lower depressive symptom scores than those living in urban areas (*B* = −3.372, *β* = −0.489, *p* < 0.001). Although participants living in urban-marginal areas and those from single-person households tended to report higher depressive symptom scores, these associations did not reach statistical significance (urban-marginal residence: *B* = 1.994, *β* = 0.289, *p* = 0.075; single-person household: *B* = 2.413, *β* = 0.350, *p* = 0.106) (see Table 3).

**Table 3:** Factors associated with depressive symptoms and self-care agency capacity among hypertensive older adults.

Variables	Self-care agency capacity							Depressive symptoms						
	Unstandardized coefficients		Standardized coefficients			95% CI lower	95% CI upper	Unstandardized coefficients		Standardized coefficients			95% CI lower	95% CI upper
	*B*	SE	*β*	*t*	*p*			*B*	SE	*β*	*t*	*p*		
Constant	62.333	2.85	—	21.882	<0.001	56.718	67.949	5.665	1.719	—	3.296	0.001	2.277	9.054
Male sex	0.112	1.47	0.01	0.076	0.939	−2.778	3.001	−2.255	0.884	−0.327	−2.550	**0.011**	−3.999	−0.512
Age 75–84 years	2.643	1.42	0.242	1.867	0.063	−0.148	5.435	0.735	0.854	0.107	0.86	0.391	−0.949	2.419
Age ≥85 years	−4.325	2.67	−0.395	−1.618	0.107	−9.594	0.944	8.593	1.613	1.246	5.328	**<0.001**	5.414	11.772
Living alone	1.581	1.67	0.145	0.944	0.346	−1.719	4.88	1.024	1.01	0.149	1.013	0.312	−0.968	3.015
Rural residence	−5.626	1.56	−0.514	−3.616	**<0.001**	−8.692	−2.559	−3.372	0.939	−0.489	−3.592	**<0.001**	−5.222	−1.521
Urban-marginal residence	8.207	1.85	0.75	4.448	**<0.001**	4.57	11.845	1.994	1.113	0.289	1.79	0.075	−0.201	4.188
Basic educational level	−3.753	2.56	−0.343	−1.464	0.145	−8.805	1.3	1.536	1.547	0.223	0.993	0.322	−1.513	4.585
Intermediate educational level	−1.125	3.19	−0.103	−0.353	0.725	−7.409	5.16	0.792	1.924	0.115	0.411	0.681	−3.001	4.584
Extended family	0.172	1.92	0.016	0.09	0.929	−3.614	3.958	1.595	1.159	0.231	1.376	0.17	−0.690	3.879
Single-parent family	−3.592	2.36	−0.328	−1.525	0.129	−8.235	1.051	0.278	1.421	0.04	0.195	0.845	−2.524	3.079
Single-person household	0.057	2.46	0.005	0.023	0.982	−4.800	4.913	2.413	1.487	0.35	1.623	0.106	−0.517	5.344

*B* = unstandardized coefficient; SE, standard error; *β* = standardized coefficient; 95% CI, 95% confidence interval. Self-care agency capacity model: *R* = 0.500; *R*^2^ = 0.250; adjusted *R*^2^ = 0.211; *F* (11, 210) = 6.36; *p* < 0.001; RMSE = 9.45. Depressive symptoms model: *R* = 0.559; *R*^2^ = 0.313; adjusted *R*^2^ = 0.277; *F* (11, 210) = 8.68; *p* < 0.001; RMSE = 5.70. Reference categories were female sex, married marital status, urban residence, nuclear family structure, age 65–74 years, and higher educational level. Bold values indicate statistical significance with *p* < 0.05.

## 4 Discussion

This study examined the relationship between SCAC and depressive symptoms among community-dwelling older adults with HTN living in Loja Province, Ecuador, while also exploring the influence of sociodemographic characteristics on both outcomes. The main findings showed that no statistically significant association was identified between SCAC and depressive symptoms. In contrast, place of residence remained independently associated with SCAC, whereas sex, age, and place of residence were independently associated with depressive symptoms.

Although previous studies have consistently reported an inverse relationship between depressive symptoms and self-care among individuals with chronic diseases (14, 20, 34, 35), the present findings suggest that this association may not be universal but rather influenced by contextual, psychological, and healthcare-related factors.

One possible explanation for the absence of this association is that most participants presented minimal depressive symptoms together with high levels of SCAC, limiting the variability required to detect statistically significant correlations. Moreover, all participants were receiving regular follow-up through primary healthcare services, which may have reinforced self-care practices regardless of their emotional status. Consequently, depressive symptoms and SCAC may not necessarily evolve in parallel among community-dwelling older adults with HTN. This interpretation is supported by studies indicating that the relationship between psychological distress and self-care is often mediated by factors such as self-efficacy, perceived social support, access to healthcare services, and participation in structured disease-management programs rather than by depressive symptoms alone (14, 34–36).

The present findings may also be interpreted in light of Dorothea Orem’s Self-Care Deficit Nursing Theory, which proposes that SCAC is determined by Basic Conditioning Factors, including age, environmental context, available resources, health status, and sociocultural conditions. From this perspective, self-care is understood as a multidimensional capability influenced not only by individual motivation but also by the environmental and social conditions that facilitate or hinder its performance. Accordingly, the lack of an independent association between depressive symptoms and SCAC observed in this study suggests that contextual determinants may exert a stronger influence on self-care than emotional status alone. This interpretation is further supported by the finding that place of residence remained the only independent predictor of SCAC after adjustment, reinforcing Orem’s proposition that environmental conditions are fundamental determinants of individuals’ capacity to maintain self-care behaviors (17, 37).

Advanced age and female sex were independently associated with higher depressive symptom scores, consistent with previous evidence indicating that aging, multi-morbidity, functional decline, and biological as well as psychosocial factors increase vulnerability to depression among older adults, particularly female participants (9, 11, 38). These findings are also consistent with evidence demonstrating an association between hypertension and depression among older adults (39). Therefore, routinely assessing mental health in hypertensive older adults, especially among female participants and the oldest age groups, may facilitate the early identification of depressive symptoms and timely interventions, consistent with current recommendations supporting depression screening in primary care (40).

Another relevant finding was the influence of place of residence on SCAC. Loja Province is characterized by considerable geographic and social heterogeneity, with substantial differences in healthcare accessibility, transportation, educational opportunities, and community resources across urban, rural, and urban-marginal settings. These contextual characteristics may partly explain why rural residence was associated with lower SCAC, whereas participants living in urban-marginal areas demonstrated higher self-care scores. Older adults residing in rural communities often face greater barriers to healthcare access, health education, and continuity of chronic disease management, while those living closer to primary healthcare facilities may benefit from more frequent professional support and health promotion initiatives. Previous studies have similarly demonstrated that environmental conditions, territorial inequalities, social support, and equitable access to healthcare services are key determinants of self-care and chronic disease management among older adults with HTN and other long-term conditions (20, 35, 41, 42).

Although older adults living alone exhibited higher depressive symptom scores in the bivariate analyses, this association was not maintained after multivariable adjustment, suggesting that the apparent effect of family structure may be explained by other sociodemographic characteristics. Nevertheless, extensive evidence indicates that family and social support remain important protective factors against psychological distress by strengthening emotional coping, promoting treatment adherence, and facilitating engagement in self-care activities among older adults with chronic diseases (43, 44).

Likewise, participants with higher educational attainment demonstrated greater SCAC in the descriptive analyses; however, this association was not retained in the adjusted regression model. Although educational level is frequently associated with higher health literacy and a better understanding of therapeutic recommendations, the present findings suggest that environmental and contextual conditions may have a greater influence on SCAC than education alone in this population. Nevertheless, recent evidence indicates that interventions aimed at improving health literacy enhance self-efficacy, medication adherence, and long-term self-management among older adults with HTN, highlighting health literacy as an important component of comprehensive HTN care (45–48).

### 4.1 Implications for aging and public health

The findings of the present study highlight the importance of incorporating mental health assessment and the social determinants of health into the comprehensive management of older adults with HTN, particularly within primary healthcare settings. Providing care that extends beyond blood pressure control to include the identification of depressive symptoms and contextual vulnerabilities may contribute to a more comprehensive understanding of the needs of aging populations living in socially and territorially heterogeneous environments (20, 46).

Likewise, strengthening self-care through health education, health literacy, and community support interventions may contribute to improved disease management and promote healthy aging among older adults with chronic conditions (45, 49). In this context, primary healthcare services play a fundamental role in the early identification of psychosocial vulnerabilities and the implementation of person-centered interventions adapted to territorial and social realities (35).

This study also contributes to the limited evidence available in Ecuador regarding the simultaneous analysis of depressive symptoms, SCAC, and sociodemographic determinants among older adults with HTN. These findings may inform future public health strategies aimed at reducing health inequalities and strengthening comprehensive care for aging populations in low- and middle-income settings, particularly through community-based and context-sensitive approaches to healthy aging (46, 50, 51).

### 4.2 Limitations

The present study has several limitations that should be considered when interpreting the findings. First, the cross-sectional design precludes establishing causal relationships between sociodemographic factors, depressive symptoms, and self-care agency capacity. In addition, data were collected using self-report instruments, which may be subject to recall and social desirability bias.

Although participants from urban, rural, and urban-marginal areas were included, the sample was recruited from a single province in southern Ecuador, limiting the generalizability of the findings to other populations. Furthermore, some sociodemographic categories, particularly religion and ethnicity, included relatively small numbers of participants, which may have reduced the stability of the statistical estimates. The use of non-probability convenience sampling may also have introduced selection bias and limited the representativeness of the sample.

Finally, clinically relevant variables, including duration of arterial hypertension, antihypertensive medication use, treatment adherence, blood pressure control, and the presence of comorbidities, were not assessed, although these factors may influence both depressive symptoms and self-care agency capacity. Future longitudinal studies incorporating these variables together with objective blood pressure measurements are needed to provide a more comprehensive understanding of these relationships.

## 5 Conclusion

This study demonstrated that specific sociodemographic factors were independently associated with depressive symptoms and self-care agency capacity among older adults with arterial hypertension. Place of residence remained the only independent predictor of self-care agency capacity, whereas sex, age, and place of residence were independently associated with depressive symptoms. In contrast, no statistically significant association was identified between depressive symptoms and self-care agency capacity in this population.

These findings emphasize the importance of considering social determinants and mental health within the comprehensive management of older adults with arterial hypertension. Integrating the assessment of contextual living conditions and self-care agency capacity into primary healthcare may support the development of more equitable, person-centered, and context-sensitive interventions, particularly in territorially heterogeneous settings such as Loja Province, Ecuador.

## Data Availability

The raw data supporting the conclusions of this article will be made available by the authors, without undue reservation.
