# Supplementary material for: Sociodemographic factors associated with depressive symptoms and self-care agency capacity in hypertensive older adults from Loja, Ecuador: a cross-sectional study
Source: Front Public Health. 2026 Sep 10;14:1888008. doi: 10.3389/fpubh.2026.1888008 (PMC13600824; doi:10.3389/fpubh.2026.1888008)
Supplement: Supplementary file 1 [file Supplementary_file_1.docx]

# Supplementary Material 1

Spanish versions of the instruments used during data collection.

The following instruments correspond to the original Spanish versions applied during data collection in older adults with hypertension in Loja, Ecuador.

## 1. Self-Care Agency Capacity Questionnaire in Hypertensive Patients (Original Spanish Version)

The following questionnaire was applied in its original Spanish version as part of the assessment of self-care agency capacity.

| Ítem | Pregunta |
| --- | --- |
| 1 | Cuando duermo 6-8 horas diarias, me siento descansado. |
| 2 | Invierto tiempo en mi propio cuidado. |
| 3 | Considero importante realizar ejercicio. |
| 4 | Busco información y orientación sobre el manejo de mi enfermedad. |
| 5 | Considero que conozco cómo actúan y las reacciones desfavorables de los medicamentos que tomo actualmente para controlar la presión arterial. |
| 6 | Cuando inicio un nuevo medicamento solicito al profesional de enfermería me proporcione información necesaria de la correcta administración. |
| 7 | Considero que la cantidad máxima de sal que puedo utilizar para todo el día en la preparación y consumo de alimentos son 2 cucharaditas de sal. |
| 8 | Reemplazo mi tratamiento por terapias alternativas. |
| 9 | Considero que el profesional de enfermería me escucha y aclara mis inquietudes. |
| 10 | Conozco las complicaciones derivadas de la enfermedad. |
| 11 | Camino diariamente como mínimo 30 minutos. |
| 12 | Realizo cambios según las necesidades para mantener la salud y manejar adecuadamente la enfermedad. |
| 13 | Realizo cambios en mi dieta para mantener un peso adecuado. |
| 14 | Evalúo si las medidas que he tomado en el manejo de mi enfermedad permiten garantizar mi bienestar y el de mi familia. |
| 15 | Consumo en mi dieta comidas ricas en verduras, frutas, fibra y baja en grasas. |
| 16 | He tenido dificultades con el acceso a las consultas, controles y adquisición de medicamentos. |
| 17 | Siento que mi enfermedad ha generado dificultades en el desarrollo adecuado de mi vida sexual. |

Response options ranged from 1 (Nunca) to 5 (Siempre). Negative items (8, 16, and 17) were reverse coded during statistical analysis.

## 2. Beck Depression Inventory-II (BDI-II)

The Beck Depression Inventory-II (BDI-II) was applied in its validated Spanish version.

Due to copyright restrictions, the complete instrument is not reproduced in this supplementary material.

## 3. Ethical Considerations

The instruments included in this supplementary material are presented exclusively for academic and scientific purposes as part of the transparency and reproducibility process of the present study.
